# ACO (Asthma–COPD Overlap) Is Independent from COPD, a Case in Favor: A Systematic Review

**DOI:** 10.3390/diagnostics11050859

**Published:** 2021-05-11

**Authors:** Naoya Fujino, Hisatoshi Sugiura

**Affiliations:** Department of Respiratory Medicine, Tohoku University Graduate School of Medicine, Sendai 980-8574, Japan; sugiura@rm.med.tohoku.ac.jp

**Keywords:** asthma–COPD overlap, asthma, COPD, fractional exhaled nitric oxide, immunoglobulin E

## Abstract

Asthma and chronic obstructive pulmonary disease (COPD) are now recognized to be able to co-exist as asthma–COPD overlap (ACO). It is clinically relevant to evaluate whether patients with COPD concurrently have components of asthma in primary care. This is because: (i) ACO is a relatively common condition among asthma (over 40 years of age) or COPD irrespective of its diagnosis criteria; (ii) patients with ACO can have higher frequency of exacerbation and more rapid decline in lung function than those with asthma or COPD; and (iii) asthmatic features such as eosinophilic airway inflammation are promising indicators for prediction of inhaled corticosteroid-responsiveness in COPD. The aim of this review to evaluate diagnostic markers for ACO. We searched PubMed for articles related to ACO published until 2020. Articles associated with diagnostic biomarkers were included. We identified a total of 25 studies, some of which have revealed that a combination of biomarkers such as fractional exhaled nitric oxide and serum immunoglobulin E is useful to discern type 2 inflammation in the airways of COPD. Here, we review the current understanding of the clinical characteristics, biomarkers and molecular pathophysiology of ACO in the context of how ACO can be differentiated from COPD.

## 1. Introduction

### 1.1. Background of Asthma and Chronic Obstructive Pulmonary Disesase

The global burdens of asthma and chronic obstructive pulmonary disease (COPD) are increasing, each of which was estimated to affect respectively approximate 339 million and 251 million people worldwide in 2016 [1]. It has been widely accepted that asthma and COPD are strikingly different airway disorders [2,3]. Although the “Dutch hypothesis” suggested a common genetic background underlying airway obstruction with a spectrum of clinical entities from asthma to COPD, recent genetic research indicated that it was unlikely that genetic factors are shared by asthma and COPD [4].

Asthma is a heterogenous and inflammatory disease affecting large and small respiratory tracts but not the lung parenchyma, and contains clusters of demographical, clinical and pathophysiological characteristics underpinned by different pathophysiological processes [5]. This heterogeneity may be explained by the complexity of dysregulated innate and adaptive inflammatory responses to exogenous allergens and proteases leading to the spectrum of abnormal tissue remodeling, where type 2 cytokines such as interleukin (IL)-4, IL-13 and IL-5 primarily promote airway eosinophil infiltration, mucus hypersecretion, bronchial hyperresponsiveness and mast cell activation [6]. Major subpopulations of asthmatics have molecular signatures of T helper 2 (Th2)—inflammation and airway obstruction that markedly respond to inhaled corticosteroid (ICS) [7]. In line with this translational study, accumulated evidence from randomized control trials have revealed the importance of ICS usage from the early steps of asthma treatment because clinical studies have shown that ICS robustly reduced the risk of symptoms, exacerbations, hospitalization and mortality from asthma [8,9,10].

COPD is defined as a common, preventable and treatable disease that is characterized by persistent respiratory symptoms and airflow limitation that is due to airway and/or alveolar abnormalities usually caused by significant exposure to noxious particles or gases and influenced by host factors including abnormal lung development [11]. In addition to cigarette smoking, known as the most common COPD risk factor [12], the susceptibility could be influenced by genetic factors [13,14] and abnormal lung growth [15]. Unlike asthma, CD4^+^ T helper 1 (Th1) cells, CD8^+^ cytotoxic T (Tc) cells, neutrophils and macrophages predominantly affect the small airways and the lung parenchyma leading to mucus hypersecretion, alveolar wall destruction (emphysema) and small airway fibrosis in COPD [2,16]. These pro-inflammatory cell-types are functionally altered by oxidative stress and intracellular signaling pathways including activation of the proinflammatory transcription factor nuclear factor κB (NF-κB) [17], and alveolar macrophages are defective in bacterial phagocytosis, possibly via several phagocytic receptors and mitochondrial molecules related to oxidative stress [18,19,20,21]. The small airway narrowing induced by pro-inflammatory cell infiltration, luminal exudates, wall thickening, and the loss of small airways associated with emphysema increases airway obstruction [22,23]. In the wall thickening, hyperplasia of basal cells, known as airway epithelial stem cells, could be formed through several molecules such as Axl receptor tyrosine kinase [24] and Yap-Wnt7b [25]. The airflow limitation progressively leads to gas-trapping in peripheral lungs during expiration on exercise, resulting in dynamic hyperinflation which is postulated to be the main mechanism of exertional dyspnea [26,27]. Thus bronchodilators, long-acting muscarinic antagonists (LAMA) and long acting beta_2_-agonists (LABA), are commonly used as the pharmacological therapy for COPD and are known to reduce lung hyperinflation, dyspnea and exercise endurance [28,29] leading to improvement of the quality of life and a reduction in the frequency of exacerbations [30]. Accumulated evidence indicates that LAMA significantly reduce the frequency of exacerbations and non-serious adverse events and increase the trough forced expiratory volume in one second (FEV_1_) compared to LABA in patients with stable COPD [31].

### 1.2. Safety Issues of ICS for Airway Infection of Patients with COPD

Several lines of evidence have indicated a higher risk of pulmonary infection in COPD patients. A population-based, case-control study conducted in Spain including 859,033 inhabitants showed a strong relationship between COPD and community-acquired pneumonia, which was independent of other clinical factors (odds ratio (OR) 1.84 (95% confidence interval (CI), 1.32–2.59)) [32]. A prospective case-control study with 175,906 COPD subjects in Canada also demonstrated that current use of ICS further increased the risk of hospitalization for pneumonia (rate ratio (RR) 1.70 (95% CI, 1.63–1.77)) and pneumonia followed by death within 30 days (RR 1.53 (95% CI, 1.30–1.80)) [33]. Particularly, a subset of COPD treated with ICS who had both less than 100 cells/μL of blood eosinophils and chronic bronchial infection by potentially pathogenic microorganisms was at higher risk of pneumonia (OR 3.238 (95% CI, 1.426–7.231)) [34]. Moreover, the current use of ICS in subjects even without oral corticosteroid also increased the risk of tuberculosis (TB) in a low-prevalence country (RR 1.33 (95% CI, 1.04–1.71)) [35] as well as in an intermediate-burden setting (OR 1.20 (95% CI 1.08–1.34)) [36]. In addition, the increased risk of TB infection was significantly associated with higher doses of ICS [35,36]. These studies highlighted the importance of the risk of pulmonary infection among COPD patients who are treated with high dose ICS and provided the clinically relevant question of which subset of COPD subjects would benefit from ICS therapy.

### 1.3. Needs for Considering Patients with Clinical Features of Both Asthma and COPD

From the early 2000s onwards, the differential diagnosis of patients with respiratory symptoms who are more than forty years of age has been recognized to be more problematic. This is because COPD becomes more common in older adults and discriminating asthma with persistent airflow obstruction from COPD is often challenging [37,38]. In fact, in 2007, the *Canadian Thoracic Society Recommendations for Management of Chronic Obstructive Pulmonary Disease—2007 Update* described the concept of “combined COPD and asthma” to highlight the finding that early introduction of ICS could be justified if the COPD patients had prominent features of asthma [39]. In 2009, Gibson and Simpson introduced the word of “the overlap syndrome of asthma and COPD” and noted that, since these patients had been largely excluded from pivotal therapeutic trials for both asthma and COPD, its diagnosis and treatment were poorly defined and lacking firm evidence [40]. Despite a growing controversy over clinical and prognostic features of ACO, retrospective studies have provided the plausible premise showing that patients with both asthma and COPD have more respiratory symptoms [41], high frequency of exacerbations [41,42] and accelerated decline in lung function [43]. Thereafter, articles regarding asthma–COPD overlap syndrome (ACOS) have been widely reviewed [44,45,46]. Following these early reviews, in 2014, the Global Strategy for Asthma Management and Prevention (GINA) and the Global Initiative for Chronic Obstructive Pulmonary Disease (GOLD) jointly issued their consensus-based document on ACOS so that clinicians were able to distinguish asthma from COPD and make a diagnosis of ACOS in patients with chronic airflow limitation [47]. GINA then suggested the descriptive term asthma–COPD overlap (ACO) rather than ACOS, which had been often interpreted as implying a single disease [48].

In 2016, based on the urgent requirement of an operational definition of ACO, a global expert panel from North America, Western Europe and Asia proposed criteria for its diagnosis, which consisted of age, smoking history, the presence of persistent airflow limitation defined by spirometry, history of asthma and allergic rhinitis, bronchodilator response in FEV_1_ and peripheral blood eosinophil counts [49]. Following this attempt, some guidelines, such as Spanish [50] and Japanese [51] guidelines, published diagnostic algorithms that employed objective evaluations of eosinophilic and allergic airway inflammation by quantifying the levels of fractional exhaled nitric oxide (FeNO) and serum immunoglobulin E (IgE). However, the GOLD 2020 update has stated that it no longer mentions ACO [11]. This was because asthma and COPD were different disorders, although they might share common traits and clinical features. In addition, it further documented that “if a concurrent diagnosis of asthma is suspected, pharmacotherapy should primarily follow asthma guidelines, but pharmacological and non-pharmacological approaches may be needed for their COPD”. This has given rise to pro and con arguments for experts in obstructive lung diseases [52,53]. As patients with ACO have been certainly excluded from clinical studies of asthma and COPD [49], molecular mechanisms of the disease and evidence of appropriate therapies are less well understood. Considering the pro-con discussion, we still have a large, unresolved question concerning why we should have a diagnostic term of ACO and how we can distinguish between COPD and ACO. The rationale of this systematic review is to describe how ACO differs in clinical features and pathogenesis from COPD. Thus, we aimed to elaborate clinical features and diagnostic markers that enable to discriminate ACO from COPD.

## 2. Methods

### 2.1. Search Strategy and Eligibility Criteria

This systematic review adheres to the PRISMA guidelines [54]. We searched PubMed for publications until 2020 with terms of “asthma–COPD overlap” or either “diagnosis” or “biomarker”. Articles were included if they indicated a total population to evaluate diagnostic biomarkers such as FeNO, IgE, humoral factors or radiographical findings to be able to differentiate ACO from COPD. We excluded articles that: (1) were not associated with diagnosis of ACO by the titles and abstracts; (2) were systematic reviews or guidelines; (3) were subset analyses in other studies; (4) did not report any biomarkers except for blood eosinophil counts and pulmonary function tests. We finally added five articles reporting FeNO-driven identification of ICS responders in patients with COPD as citation searching.

### 2.2. Data Collection and Risk of Bias Assessment

Two review authors (NF and HS) screened the titles and abstracts of all studies identified. Full text assessments were performed to identify studies that met inclusion criteria. The risk of bias in the eligible studies was evaluated in accordance with the recommendations in the Cochrane Handbook for Systematic Reviews of Interventions 5.1.0.

## 3. Results

### 3.1. Characteristics of Selected Studies and Risk of Bias

**Figure 1:**
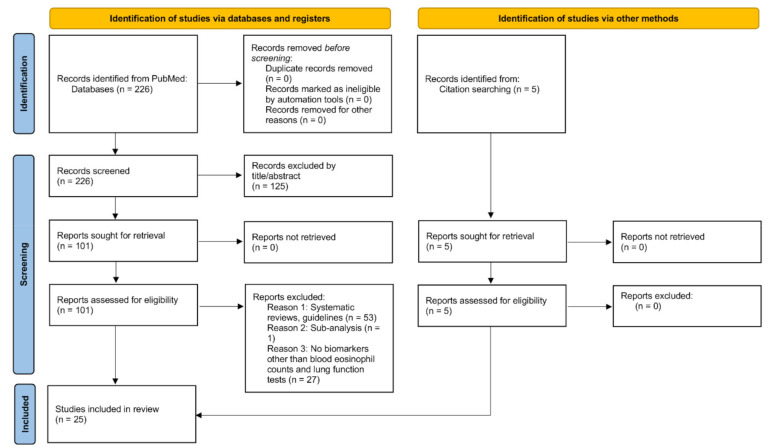
Study selection.

The literature search yielded 226 candidate studies. After excluding studies on the basis of their titles or abstracts or through examining their full texts, 20 were identified. We also included five articles reporting FeNO-driven identification of ICS responders in patients with COPD as citation searching. Thus, 25 studies were included in this review (Figure 1). These studies were summarized in Table 1. Diagnostic markers for ACO included FeNO, combination of FeNO and IgE, blood, urine or induced sputum biomarkers such as inflammatory cytokines and radiographical parameters (Table 1). There were high selection bias and performance bias in the included studies.

### 3.2. Possible Diagnostic Biomarkers for ACO: The Role of Fractional Exhaled Nitric Oxide, FeNO and IgE for the Detection of Asthmatic Features of COPD Patients

#### 3.2.1. FeNO as a Potential Biomarker for Type 2 Inflammation for Optimal Diagnosis and to Predict the Treatment Response in Asthma.

Nitric oxide (NO) mainly originates from respiratory epithelial cells and is dominantly produced through inducible NO synthase (iNOS). Homeostatic interferon (IFN)-γ and its downstream molecule, signal transducer and activator of transcription (STAT)-1, maintains iNOS expression in the airway epithelium of healthy subjects [55]. Alving et al. firstly reported level that the FeNO of patients with mild atopic asthma was two- to three-fold higher than that of healthy control subjects [56]. A large-scale general population study supported this preliminary data by demonstrating that individuals with both increased FeNO levels and blood eosinophil counts had an increased risk of respiratory symptoms of asthma and ACO [57]. In addition, the FeNO levels were significantly increased in atopic asthma compared to non-atopic asthma [58]. The increase in the FeNO levels was underpinned by the up-regulation of iNOS mRNA and protein expression in the airway epithelium of asthmatics [59,60,61,62]. A clinical study using a selective iNOS inhibitor further confirmed that up-regulation of the FeNO levels dominantly depended on the increased iNOS expression in asthmatic patients [63]. The extent of FeNO was significantly correlated with the eosinophil counts in induced sputum [64], endobronchial biopsies [65] and bronchoalveolar fluid [66] in asthmatics, suggesting that FeNO could be used as a surrogate marker for eosinophilic airway inflammation. Although airway hyperresponsiveness (AHR) of steroid-naïve asthmatics at baseline was not associated with airway inflammation markers such as FeNO and eosinophils in induced sputum, the improvement of AHR and FEV_1_ by inhaled corticosteroid therapy was significantly correlated with a reduction in the FeNO levels in those patients [67]. Researchers and clinicians in the field of asthma and allergy have given much attention to the molecular mechanisms underlying the upregulation of iNOS in asthmatic airways. Two groups reported that IL-13 induced iNOS mRNA and protein expression, which was significantly correlated with NO gas production in primary bronchial epithelial cells from healthy subjects [68] and mild-moderate asthmatics [69]. The contribution of the IL-4 and IL-13 pathway to the increase in airway NO production was further confirmed by clinical trials demonstrating that the FeNO levels were reduced in asthmatic patients treated with IL-4/IL-13 signaling blockade including nebulized soluble recombinant IL-4 receptor [70], inhaled recombinant IL-4 variant [71] and a monoclonal antibody to the alpha subunit of the IL-4 receptor alpha [72]. Collectively, these basic, translational and clinical studies have shown convincing evidence that FeNO could be a surrogate marker for type 2 inflammation of the airway.

#### 3.2.2. How Can Feno Be Adopted to Discern ICS-Responsive, Asthmatic Phenotypes in COPD?

COPD is a highly complex and heterogenous disease, including several characteristics that could provide a rationale for the development of precision medicine [73]. Because inappropriate treatment with ICS is known to increase the risk of pneumonia for COPD patients, as discussed above, ICS should be ideally used for patients who can be expected to respond to corticosteroid therapy. Brightling et al. performed a randomized, double blind, crossover trial revealing that subjects with COPD who had higher eosinophil counts in their induced sputum exhibited increased post-bronchodilator FEV_1_ after six-week-treatment with ICS [74]. Based on this research, much effort has been given to ask whether FeNO can be used to identify asthma-associated features related to a favorable ICS response among COPD patients (Table 1). These studies, despite the small numbers of patients included, produced consistent and substantial results demonstrating that the initial FeNO levels were significantly correlated with the improvement of airway obstruction evaluated by FEV_1_ or ΔN_2_ after additional ICS therapy [75,76,77,78,79]. Moreover, a recent double-blind randomized placebo-controlled trial including 214 undiagnosed subjects who had cough, wheeze or dyspnea showed that FeNO could be used in clinical practice for patients with non-specific respiratory symptoms in order to predict the ICS response [80]. Although these studies showed promise for the general use of FeNO in the clinical setting of COPD, it is still unclear whether a FeNO cut-off value could be determined to identify an ICS-responsive subset of COPD patients [81]. This question is of particular importance because the GOLD guideline limited the use of ICS to only the following types of patients: (i) Group D patients with greater than 300 cells/μL of blood eosinophils in initial pharmacological treatment or in follow-up pharmacological management post exacerbations and (ii) patients with more than 100 cells/μL of blood eosinophils when experiencing more than 2 moderate exacerbations per year or at least one severe exacerbation requiring hospitalization in the prior year [11]. Recent clinical studies compared FeNO levels between COPD and ACO and confirmed high accuracy of diagnosis to discriminate ACO from COPD [82,83,84,85,86].

#### 3.2.3. Serum IgE Elevation and Atopic Background for COPD and ACO

Atopy refers to a genetic tendency to produce IgE typically for common and environmental allergens including inhaled allergens and food allergens. The presence of IgE specific for inhaled allergens, such as dust mites, cockroach and animal dander, is known to be a risk factor for asthma [102,103]. Serum total IgE levels were significantly correlated in pairs of siblings for bronchial hyperresponsiveness to histamine, which is implicated as a coinherited trait [104]. In addition, symptoms and exacerbations of asthma were significantly associated with an elevation of serum total IgE, which was independent of specific IgE [105]. This intriguing role of total, non-specific IgE in the association with asthma may be explained by the ability of IgE to enhance mast cell survival via Fcε receptor I cross-linking [106]. Serum levels of total IgE, IL-4 and leukotriene B4 were increased in ACO compared to COPD [107]. These observational and translational studies suggest that the mast cell activation pathway is upregulated in ACO as with asthma. This hypothesis was further supported by at least two clinical studies indicating that omalizumab, a humanized monoclonal antibody for IgE, markedly improved asthma control and health-related quality of life in ACO and this observed improvement in ACO was as large as that in asthma [108,109]. Given the evidence regarding IgE biology in atopic asthma overlapping COPD, it may be sensible to evaluate asthma complications in COPD patients with atopy.

#### 3.2.4. Combination of Type 2 Inflammation-Related Biomarkers to Define ACO

In order to maximize the benefits of ICS for COPD patients while preventing them from having pulmonary infectious diseases including pneumonia and TB caused by its inappropriate use, a combination of biomarkers may be promising for precisely defining an ICS-responsive subset. Based on this concept, our group attempted to determine whether the combination of FeNO and serum IgE had the ability to discern asthmatic, ICS-responsive features among patients with COPD. We found that the sensitivity was 1.0 and the specificity was 0.56 for the subjects who had reversibility of airway obstruction by 12-week-inhalation therapy of fluticasone propionate (FP)/Salmeterol (SAL) when FeNO > 35 parts per billion (ppb) and/or a positive result for specific IgE (atopy+) were combined [78]. Especially, all of the patients with both FeNO > 35 ppb and atopy+ responded to the additional FP/SAL therapy [78]. In addition, no patients with both less than 35 ppb of FeNO and a negative result of specific IgE responded to FP/SAL. We further reported that the prevalence of expected high ICS responders (i.e., FeNO > 35 ppb and atopy+) was 7.8 % of Japanese COPD subjects, whereas that of patients who were not likely given the benefits of ICS (i.e., FeNO ≤ 35 ppb and atopy−) was 54.8% [87]. Another Japanese cohort confirmed this result [88]. These clinical proof-of-concept studies may illustrate that a combination of biomarkers related to type 2 inflammation could be useful for a more precise definition of ACO. Given this concept, in 2018 the Japanese Respiratory Society (JRS) issued guidelines for new definitions and diagnostic criteria of ACO (Table 2) [51,110]. It is noteworthy that these two biomarkers (FeNO and IgE) were included in the criteria to identify the features of asthma among COPD subjects.

Two recent reports used the criteria to better characterize the clinical features of ACO in the Japanese population. A retrospective study investigating 170 subjects with persistent airflow limitation indicated that the prevalence of ACO among COPD patients was 31.5% [111]. However, one of the limitations in the retrospective study was that the use of ICS was not controlled, and that the characterization of clinical phenotypes could be biased or masked by ICS. More recently, Hirai, et al. investigated the prevalence of ACO among ICS-naïve patients with COPD and compared the baseline characteristics between COPD and ACO [112]. 197 patients with COPD were included in this study and 38 (19.3%) met the ACO diagnostic criteria by the Japanese guidelines. Although they did not find statistical significance in the baseline clinical features (age, gender, pack years of smoking and pulmonary function) between ACO and COPD, symptoms and dyspnea evaluated by COPD assessment test (CAT) and modified MRC (mMRC) scale were significantly worse in ACO compared to COPD [112]. In addition, the frequency of acute exacerbations during the one year prior to the participation was three times higher in ACO than in COPD [112]. These studies with other literature [82,83,84,85,86] support the concept that the diagnostic criteria using biomarkers such as FeNO and IgE could be a landmark in facilitating a secure identification of ICS-responders among COPD patients. However, further worldwide research will be necessary to uncover how much the ACO diagnostic criteria could improve the clinical practice for COPD and ACO and to what extent the use of ICS could be avoided. Major problems regarding the development of diagnostic biomarkers include how biological parameters are weighed and how the cut-off values are determined. Leading-edge approach using omics and machine learning may solve this problem in an unbiased way [113,114,115].

### 3.3. Possible Biomarkers Relevant to Eosinophilic and Neutrophilic Inflammation in ACO

Christenson, et al. revealed transcriptome of airway epithelial cells in steroid-naïve subjects with mild to moderate asthma (n = 62) and control subjects without asthma (n = 43) and defined the asthma-derived gene expression signatures of Th2 inflammation [116]. The Th2-high signatures identified a clinically relevant subgroup of COPD that had a favourable response to ICS [116]. Eosinophils activated by the Th2-related priming agents, such as IL-5, IL-3, IL-33, granulocyte-macrophage colony-stimulating factor (GM-CSF) or Notch ligands acquire cell motility, increase respiratory burst and release granule proteins including eosinophil-derived neurotoxin (EDN) [117]. The serum EDN levels are known to correlate with the persistent airflow limitation with adult asthma and to be significantly decreased post eight-week treatment of omalizumab [118]. Serum EDN was significantly higher in ACO compared to asthma and COPD [119]. In addition, using a putative diagnosis biomarker for COPD, YKL-40 [120,121], combined assessment of serum EDN and YKL-40 revealed that a subset with both high serum EDN and YKL-40 levels was 45% in ACO, 14% in asthma and 30% in COPD (OR 3.85 (95% CI, 2.35–6.36); sensitivity, 45.2%; specificity 82.4%) [119]. Interestingly, this study showed other subsets of ACO based on the levels of EDN and YKL-40 (27% in high EDN and low YKL-40, 16% in low EDN and high YKL-40 and 12% in low EDN and low YKL-40), confirming that ACO was a heterogenous condition including different forms of airway diseases [47]. Since YKL-40 alone might not be reproducible for the diagnosis of ACO [93,94], combinatory evaluation might be useful.

Not only the eosinophil-related protein EDN but also a neutrophil-associated protein may be involved in the pathogenesis of ACO. Neutrophil gelatinase-associated lipocalin (NGAL) is constitutively expressed by neutrophils and has a pivotal role in antimicrobial immunity by binding bacterial siderophores and depriving bacterial iron [122]. The NGAL protein levels were elevated in induced sputum [123] and plasma [124] in patients with COPD. Moreover, the NGAL levels in bronchoalveolar lavage fluid were elevated even in subjects who had emphysematous changes with normal FEV_1_ [125,126]. Considering a biochemical study demonstrating that NGAL prevented matrix metalloproteinase (MMP)- 9 from being degraded and maintained the MMP-9 enzymatic activity [127], NGAL has been recognized to contribute to the development of pulmonary emphysema. However, the NGAL levels in induced sputum were significantly higher in ACO compared to COPD and asthma [128]. This might be explained by recent data indicating that NGAL is also secreted from epithelial cells of renal, intestinal and respiratory systems and is now considered to be a biomarker for acute kidney injury [122]. These data suggest that neutrophilic inflammation, emphysematous changes and epithelial injuries might contribute to the elevated levels of NGAL in induced sputum from patients with ACO [128]. However, pro-inflammatory cytokines associated with type 1 and type 2 immunity in peripheral blood might not be useful to diagnose ACO from COPD [91,96].

Recent studies using novel technologies have identified candidate molecules for biomarkers of ACO. Cai, et al. reported that several eicosanoids associated with allergic inflammation were upregulated in ACO compared to COPD and demonstrated high sensitivity and specificity to differentiate ACO from COPD [95]. Urine metabolomics approach found that L-histidine levels were significantly higher in ACO compared to COPD and asthma [97]. Computed tomographic analyses have captured characteristics of allergic airway inflammation in the upper and lower airways to predict the presence of asthma-related lesions in ACO [99,100,101].

## 4. Discussion

There are important problems yet to be addressed in clinical practice for ACO and COPD: Why should we have a diagnostic term of ACO? How can we distinguish between COPD and ACO? Since inappropriate use of ICS increases the risk of respiratory infection in COPD patients, we need to maximize the ability to identify the subset of favourable response to ICS. Our systematic review showed that FeNO was useful biomarker to identify asthmatic components in patients with COPD. In addition, a combination of biomarkers such as FeNO and IgE would be useful and provide promising diagnostic criteria for ACO, which could be validated by future studies. To define more precisely the pathophysiology of ACO, it would be essential to uncover the molecular mechanisms underlying inflammation, tissue damage/repair and oxidative/nitrosative stress that can differentiate ACO from COPD. In fact, several attempts have been done to clarify eosinophilic and neutrophilic inflammation using blood, induced sputum and urine.

Because there has been no global consensus on the diagnostic criteria of ACO, its prevalence considerably depends on how it is defined [129]. However, a recent meta-analysis of 27 studies from North America, Europe and Asia reported that the prevalence of ACO was estimated to be 2.0% (95% confidence interval (CI): 1.4–2.6%) in the general population, 26.5% (95% CI: 19.5–33.6%) among patients with asthma and 29.6% (95% CI: 19.3–33.9%) among patients with COPD [130]. In line with this meta-analysis, recent epidemiological studies confirm that ACO is a common disease in primary care [131,132]. These epidemiological data highlight the clinical importance of evaluating whether patients with airflow limitation have the features of both COPD and asthma and if patients with COPD concurrently have components of asthma in primary care. Thus, further studies are required to detect the features of asthma among patients with COPD using novel biomarkers.

One biological aspect which was not described here is genetics. Genome-wide association studies in non-Hispanic whites and African-American populations (n = 3120 in COPD; 450 in ACO) indicated that the most significant single nucleotide polymorphisms (SNPs) were in or near *GPR65* on chromosome 14 [133]. *GPP65* encodes the chief acid-sensing receptor, G protein-coupled receptor 65 (GPR65), which increased the cellular viability of eosinophils in allergic airway inflammatory settings in mice [134]. Interestingly, the GWAS study for ACO and COPD failed to identify known asthma-associated genetic loci such as *ORMDL3*, *IL1RL1* and *IL4R* [135], indicating that ACO was not just an overlapping condition of asthma and COPD but was associated with a specific genetic background independent of asthma or COPD.

Another aspect of ACO is associated with redox imbalance. Our group has recently reported a key role of oxidative and nitrosative stress in the pathogenesis of ACO. We reported that excessive nitrosative stress and lower antioxidant capability were observed in neutrophils and macrophages collected from the airways of patients with ACO [136]. This redox imbalance was associated with increases in IL-8, monocyte chemotactic protein-1 (MCP-1), tumor necrosis factor (TNF)-α in induced sputum and with a prospective clinical course of higher frequency of exacerbation and more rapid decline of FEV1 in ACO subjects compared to asthmatics [136]. These current data on the role of oxidative and nitrosative stress in ACO development may have clinical implications and provide novel insights for therapeutic strategies [137]. Future basic and translational research to define the molecular and cellular phenotypes of ACO differentiating from COPD are needed.

There are several limitations in this review. First, the number of patients included in the selected studies was relatively small. Second, diagnosis of asthma was not unified over the selected studies. Third, the selected studies were not randomized control trials. Finally, we did not examine publication bias. These suggest that the risk of selection and performance bias is high in this review. This may be due to the limited studies available regarding ACO diagnosis. Future studies are required to reduce bias and to more precisely define ACO pathogenesis.

## 5. Conclusions

This review demonstrates the current clinical features and diagnostic markers that enable the differentiation of ACO from COPD and discusses possible future directions that should be addressed. In conclusion, a combination of biomarkers such as FeNO and IgE is useful for ACO diagnosis to reduce airway infections in patients with COPD.

## Figures and Tables

**Table 1:** Characteristics of included studies.

References	Study Design	Subject Numbers	Severity of Airflow Limitation	Intervention or Measurement	Results
*Studies reporting an association between baseline FeNO and improvement of airway obstruction by inhaled corticosteroid therapy*					
Zietkowski, et al. [75]	Prospective	COPD 47 (current smoker 28, ex-smoker 19) Healthy control 40 (current smoker 17, non-smoker 23)	Post-bronchodilator FEV_1_ 47.07 ± 14.55% (smoking COPD), 48.9 ± 15.3% (ex-smoking COPD)	Budesoide 800 μg/day, 8 weeks	Initial FeNO was positively correlated with an increase in post-bronchodilator FEV_1_ after ICS therapy
Kunisaki, et al. [76]	Single-arm, open-label, prospective	COPD 60 (ex-smokers)	Pre-bronchodilator FEV_1_ 35.6 ± 10.6%	Fluticasone propionate 500 μg + Salmeterol 50 μg, twice daily, 4 weeks	ICS responders (increase in FEV_1_ > 200 mL after 4 weeks ICS) have higher baseline FeNO.
Lehtimaki, et al. [77]	Single-arm, open-label, prospective	COPD 40 (current smoker 29, ex-smoker 11)	Post-bronchodilator FEV_1_ 64.6 ± 2.7% (smoking COPD), 53.3 ± 4.8% (ex-smoking COPD)	Fluticasone propionate 500 μg/day, 4 weeks	Baseline FeNO was positively correlated with changes in FEV_1_/FVC
Akamatsu, et al. [78]	Single-arm, open-label, prospective	COPD 14 with emphysema on high-resolution computed tomography (all ex-smokers)	Post-bronchodilator FEV_1_ 57.6 ± 4.4%	Fluticasone propionate 250 μg + Salmeterol 50 μg, twice daily, 12 weeks	FeNO > 35 ppb and IgE positive was correlated with airway obstruction evaluated by FEV_1_ and ΔN_2_.
Yamaji et al. [79]	Single-arm, open-label, prospective	COPD 44 (ex-smokers)	GOLD stage 1/2/3/4, n = 0/34/9/0	Ciclesonide 400 μg/day, 12 weeks	Baseline FeNO was positively correlated with changes in FEV_1_ and correlated with improvement of COPD assessment test score.
*Studies reporting FeNO for ACO diagnosis*					
Alcazar-Navarrete B, et al. [82]	Cross-sectional	COPD 103 (22 ACO), never smoker 16, healthy smoker 30, asthma 43	Postbronchodilator FEV_1_ 95 ± 19% (never smoker), 96 ± 3% (healthy smoker), 90 ± 16% (asthma), 60 ± 21% (COPD)	FeNO	FeNO AUC 0.79 with an optimal-cut off 19 ppb (sensitivity 0.68, specificity 0.75)
Goto, et al. [83]	Cross-sectional	COPD 197 (ACO 23%)	Post-bronchodilator FEV_1_ 63% (95%CI, 59–67; ACO), 60% (95%CI, 60–67; COPD)	FeNO	AUC 0.63 (95% CI, 0.54–0.72)
Chen, et al. [84]	Cross-sectional	COPD 132, asthma 500, ACO 57	FEV_1_ 50.1 ± 19.3% (COPD), 88.5 ± 19.4% (asthma), 50.1± 18.6% (ACO)	FeNO	AUC 0.78 (cut-off 22.5 ppb, sensitivity 70%, specificity 75%)
Takayama, et al. [85]	Cross-sectional	COPD 65, ACO 56	FEV_1_ 69.7 ± 21.1% (COPD), 64.9 ± 17.6% (ACO)	FeNO	AUC 0.726 (FeNO cut-off level 25.0 ppb, with 60.6% sensitivity and 87.7% specificity for steroid-naïve patients)
Guo, et al. [86]	Cross-sectional	COPD 53, ACO 53	FEV_1_ 56.0% (IQR, 48.3–66.9; ACO), 43.0% (IQR, 34.8–57.1; COPD)	FeNO	AUC 0.815 (FeNO cut-off level 25.5 ppb, sensitivity 74%, specificity 77%
*Studies reporting a combination of FeNO and IgE for ACO diagnosis*					
Tamada, et al. [87]	Cross-sectional	COPD 331 (never smoker 10, ex-smoker 257, current smoker 46, unknown 18)	FEV_1_ 61.5 ± 20.8%	FeNO and serum IgE	7.8% of participants considered as ACO (FeNO > 35 ppb + IgE > 173 IU/L).
Kobayashi, et al. [88]	Cross-sectional	COPD 257	FEV_1_ 63.1 ± 32.9%	FeNO and serum IgE	AUC 0.74 (95%CI, 0.63–0.84; cut-off 23 ppb, sensitivity 73.0%, specificity 68.2%). Combination of FeNO > 23 ppb and IgE > 434 IU/mL showed 94.1% specificity and 37.8% sensitivity.
*Studies for blood biomarkers for ACO diagnosis*					
Carpagnano, et al. [89]	Cross-sectional	10 ACO (Spanish guideline), 13 ACO (GINA guideline), 13 COPD, 14 asthma, 10 healthy subjects	FEV_1_ 72.6 ± 23.4% (ACO-Spanish), 83.6 ± 22.8% (ACO-GINA), 46.9 ± 10.7% (COPD), 88.9 ± 17.7% (asthma), 91.0 ± 6.3% (healthy)	Mitochondrial and nuclear DNA in blood cells	ACO patients showed increased mitochondrial DNA in the blood cells.
Hirai, et al. [90]	Cross-sectional	COPD 50, asthma 152	FEV_1_ 63.4% (95%CI, 43.1–82.7; COPD), 86.2% (95%CI, 69.3–97.1; asthma)	mRNA expression of *TBX21*, *GATA3*, *RORC* and *FOXP3* in peripheral blood mononuclear cells	AUC 0.94 (95%CI, 0.90–0.98; total serum IgE level > 310 IU/mL, blood eosinophil counts > 280 cells/μL, a higher ratio of TBX21/GATA3, FEV1/FVC ratio < 0.67 and smoking > 10 pack-years
Llano, et al. [91]	Cross-sectional	COPD 89, asthma 94, ACO 109	Post-bronchodilator FEV_1_ 55.1 ± 18.5% (COPD), 69.5 ± 18.9% (asthma), 58.9 ± 17.0% (ACO)	IL-6, IL-8, TNF-α, IL-13, IL-5, Periostin, IL-17, FeNO	A cutoff value of FeNO > 17 ppb showed better AUC (0.707 [0.642–0.772], p < 0.001) than the cytokines or periostin in blood
Jo, et al. [92]	Cross-sectional	COPD 60, ACO 77	Post-bronchodilator FEV_1_ 71.1 ± 15.8% (COPD), 77.6 ± 16.6% (ACO)	NGAL	NGAL levels (odds ratio, 1.72; 95%CI, 0.69–4.28; ACO vs. COPD)
Wang, et al. [93]	Cross-sectional	COPD 147, asthma 124, ACO 102, control 50	Post-bronchodilator FEV_1_ 59.0 ± 9.1% (COPD), 73.7 ± 5.5% (asthma), 70.1 ± 5.6% (ACO), 95.4 ± 7.7% (control)	YKL-40, NGAL, TSLP, periostin	YKL-40 AUC 0.71 (95%CI, 0.65–0.79), cut-off < 12.61 ng/mL, sensitivity 73.5%, specificity 67.7% for ACO vs. COPD NGAL AUC 0.75 (95%CI, 0.68–0.82), cut-off < 104.7 ng/mL, sensitivity 92.7%, specificity 58.8% for ACO vs. asthma
Shirai, et al. [94]	Cross-sectional	COPD 61, asthma 177, ACO 115	FEV_1_ 66.5% (IQR, 35.8–76.3; COPD), 91.0 (78.3–102.8; asthma), 65.0 (49.0–71.5; ACO)	YKL-40, periostin, IgE, FeNO	YKL-40 AUC 0.71 (95%CI, 0.64–0.77), cut-off 61.3 ng/mL, sensitivity 60.9%, specificity 73.4% for ACO vs. asthma Periostin AUC 0.61 (95%CI, 0.53–0.70), cut-off 55.1 ng/mL, sensitivity 59.1%, specificity 62.3%
Cai, et al. [95]	Cross-sectional	COPD 27, ACO 29, Healthy control 28	FEV_1_ 40.2 ± 6.4% (COPD), 40.6 ± 8.5% (ACO), 90.8 ± 4.6% (healthy)	Eicosanoids	15(S)- hydroxyeicosatetraenoic acid, AUC 0.96
Kubysheva, et al. [96]	Cross-sectional	COPD 58, asthma 32, ACO 57	Post-bronchodilator FEV_1_ 55.3 ± 21.2% (COPD), 69.5 ± 18.9% (asthma), 58.9 ± 17.0% (ACO)	IL-17, IL-18, TNF-α	No cytokines that were able to distinguish ACO from COPD
*Studies for urine biomarkers for ACO*					
Oh, et al. [97]	Cross-sectional	COPD 38, asthma 32, ACO 37	FEV_1_ 68.1% (IQR, 48.8–85.5; COPD), 92.3% (IQR, 79.1–103; asthma), 70.0% (IQR, 51.7–85.0; ACO)	L-histidine (identified from urine metabolomics)	Urinary l-histidine levels were significantly higher in patients with ACO than in those with asthma or COPD
*Studies for biomarkers of induced sputum differentiating ACO from COPD*					
Gao, et al. [98]	Cross-sectional	Discovery cohort: 14 never smoker, 14 healthy smoker, 24 asthma, 20 COPD, 18 ACO. Replication cohort: 22 never smoker, 40 healthy smoker, 21 asthma, 35 COPD, 17 ACO	Post-bronchodilator FEV_1_ 105.9 ± 10.6% (never smoker), 98.5 ± 15.5% (healthy smoker), 78.8 ± 14.0% (asthma), 58.3 ± 19.1% (COPD), 51.6 ± 13.7% (ACO) in the discovery cohort.	IL-13, MPO, NGAL, YKL-40, IL-6 protein levels in induced sputum	Only sputum NGAL levels could differentiate ACOS from asthma (*p* < 0.001 and *p* < 0.001) and COPD (*p* < 0.05 and *p* = 0.002) in the discovery and replication cohorts.
*Studies for radiographical analyses differentiating ACO from COPD*					
Hamada, et al. [99]	Retrospective	COPD 55, asthma 39, ACO 18	FEV_1_ 54.1 ± 12.1% (COPD), 70.0 ± 13.8% (asthma), 55.8 ± 12.4% (ACO)	Radiographical evidence of sinonasal inflammation (Lund-Mackay staging, LMS)	In patients with ACO and COPD, total and ethmoid LMS scores were significantly lower than those in patients with asthma.
Qu, et al. [100]	Cross-sectional	COPD 123, ACO 106	Post-bronchodilator FEV_1_ 54.7 ± 20.8% (COPD), 64.4 ± 15.7% (ACO)	Sagittal-lung CT measurements before and after bronchodilator inhalation	Variations of all sagittal-lung CT measurements were significantly larger in patients with ACO than in patients with pure COPD (*p* values all < 0.001)
Karatama, et al. [101]	Cross-sectional	COPD 86, ACO 43	FEV_1_ 70.3 ± 20.3% (COPD), 69.4 ± 19.0% (ACO)	3 dimensional-CT	Patients with ACO had a greater wall thickness in third- to fourth-generation bronchi, smaller airway luminal area in fifth- to sixth-generation bronchi, and less emphysematous changes than did matched patients with COPD

COPD, chronic obstructive pulmonary disease; FEV_1_, forced expiratory volume in one second; FeNO, fractional exhaled nitric oxide; ICS, inhaled corticosteroid; FVC, forced vital capacity; IgE, immunoglobulin E; GOLD, global initiative for COPD. CI, confidence interval; IL, interleukin; TNF, tumor necrosis factor; MPO, myeloperoxidase; NGAL, neutrophil gelatinase-associated lipocalin; YKL-40, chitinase-like protein; IQR, interquartile range; CT, computed tomography.

**Table 2:** Diagnostic criteria for ACO issued by the Japanese Respiratory Society [110].

Fundamental Aspects: Over 40 Years of Age, Chronic Airway Obstruction Defined By < 70% of Post-Bronchodilator FEV_1_/FVC	
[Features of COPD] At least one positive features of the followings (1, 2, 3)	(Features of asthma) Two positive features of the following 1, 2, 3 items; or at least one positive features of 1, 2, 3 plus two positive features of 4
Smoking history > 10 pack-years or equivalent exposure to air pollution	Variable in diurnal, daily or seasonal symptoms, or paroxysmal respiratory symptoms (cough, sputum, dyspnea)
2. Low attenuation area indicating emphysematous changes on HRCT	2. Past history of asthma before the age of 40 years
3. Attenuated diffusion capacity (%D_LCO_ < 80% or %D_LCO_/V_A_ < 80%)	3. FeNO > 35 ppb
	4-1 Comorbidity of perennial allergic rhinitis 4-2 Reversibility of airway obstruction (FEV_1_ > 12% and > 200 mL) 4-3 Blood eosinophil > 5% or > 300 cells/μL 4-4 Elevated serum IgE (total IgE or specific IgE for perennial inhaled allergens)

COPD, chronic obstructive pulmonary disease; FEV_1_, forced expiratory volume in one second; FeNO, fractional exhaled nitric oxide; FVC, forced vital capacity; IgE, immunoglobulin E; D_LCO_, diffusing capacity of the lung carbon monoxide; V_A_, alveolar volume.
